# Surveillance as an Option for the Treatment of Small Renal Masses

**DOI:** 10.1155/2008/705958

**Published:** 2008-08-31

**Authors:** S. Klaver, S. Joniau, H. Van Poppel

**Affiliations:** Department of Urology, University Hospital Gasthuisberg, 3000 Leuven, Belgium

## Abstract

*OBJECTIVES*. To review the natural history and biological potential of small renal masses in order to evaluate surveillance as a treatment option. *METHODS*. Literature search of Medline and additional references from non-Medline-indexed publications concerning surveillance of small renal masses. *RESULTS*. The natural history and biological potential of small renal masses can still not be unambiguously predicted at present. There seems to be no clear correlation between tumour size and presence of benign histology. The majority of small renal masses grow and the majority are cancer, but one cannot safely assume that a lack of growth on serial CT scans is the confirmation of absence of malignancy. Needle core biopsies could be used to help in decision making. They show a high accuracy for histopathological tumour type but are less accurate in evaluating Fuhrman grade. *CONCLUSIONS*. At present, surveillance of small renal masses should only be considered in elderly and/or infirm patients with competing health risks, in those with a limited life expectancy, and in those for whom minimal invasive treatment or surgery is not an option. In all other patients, active surveillance should only be considered in the context of a study protocol. Long-term, prospective studies are needed to provide a more accurate assessment of the natural history and metastastic potential of small renal masses.

## 1. INTRODUCTION

The increased use of modern imaging techniques has lead to an increase in incidentally detected small renal tumours, leading to an increase of asymptomatic small renal masses with no evidence of metastatic disease. The greatest incidence of these tumours occurs in patients older than 70 years in whom multiple comorbidities may increase the risks of surgery [1].

The accepted standard treatment for the last 50 years for clinically localised renal cell carcinoma (RCC) has been radical nephrectomy; however, more recently nephron-sparing surgery has become the gold standard for most small renal masses.

There is an increased interest in minimally invasive therapies nowadays, such as radiofrequency ablation and cryotherapy, with encouraging short-term data. However, long-term outcome data are still awaited. Despite the decreased perioperative morbidity of partial nephrectomy with advances in surgical techniques, especially the laparoscopic approach, some patients may not be candidates for surgery because of medical comorbidities [2–4] or unwillingness to undergo surgical resection.

Previous findings assume that many small renal masses have a slow growth rate and a low metastatic potential. This has raised the question of close monitoring as an alternative to surgery. To be able to implement such strategy, a better understanding of the biological behaviour and natural history of these lesions is important. We reviewed the available data from two prospective studies [5, 6] and several small case series [7–17] describing the short-term outcomes of expectantly followed renal masses to better assess the current role of active surveillance as a therapeutic strategy of these small renal masses.

## 2. SMALL RENAL MASSES AND THEIR NATURAL HISTORY

Kouba et al. [1] suggested that active surveillance for renal masses is an appropriate option in selected patients, especially those with competing comorbidities, since delayed intervention after an initial period of surveillance does not appear to adversely impact pathological outcomes. They published the results of a retrospective case series including 43 patients with 46 renal masses (24% of tumours >4 cm) who underwent active surveillance of enhancing solid or cystic Bosniak IV renal masses. A subset of 13 patients who ultimately underwent surgical intervention was also examined. Mean delay to intervention was 12 months. At a 36 months mean follow-up, renal masses grew in 74% of patients with a mean (median) growth rate of 0.70 (0.35) cm per year, no patient died of RCC and none had evidence of metastatic disease. Initial tumour size (3.1 versus 2.6 cm, *p* = 0.4504) was similar in the intervention and nonintervention groups and growth rate did not correlate with initial tumour size.

Volpe et al. [5] reported in their report that only one third of small renal masses grew during surveillance and therefore raising the possibility of a period of initial observation in selected patients, especially in infirm or elderly patients. They prospectively followed 29 patients with 32 small renal masses (<4 cm, 7 lesions were complex cystic masses) for a median of 27.9 months who refused or were deemed unfit for surgical treatment. The median baseline volume was 4.9 cm^3^ for the 25 solid masses and 22.8 cm^3^ for the 7 cystic masses. The average growth rates for the solid and the cystic masses were comparable (0.11 and 0.09 cm per year, resp.; *P* = .41).

Also Rendon et al. [6], who prospectively followed 13 patients with small renal masses for a median of 42 months, concluded that most small renal masses grow slowly, if at all, and metastases are unlikely to arise before the mass shows rapid growth. Later, in 2006 Rendon and Jewett [18] reviewed the available data on the natural history of small renal masses and considered surveillance a feasible and safe option in patients with a short-life expectancy or within a well-controlled clinical trial. They recommended close imaging follow-up should be performed every 3 months for the 1st year, every 6 months for the next 2 years, and every year thereafter. A cut-off tumour size of 4 cm was considered safe although this has not yet been systematically validated.

Most other published series concerning this matter are small retrospective case series with limited follow-up and pathology data for many patients are missing [7–15]. The individual investigators advise that a period of surveillance can be safely performed in patients who are medically unfit for surgery.

Chawla et al. [16] performed a meta-analysis in which they combined the data from several small observational series and their institutional series, including 234 untreated localised small renal masses with a mean follow-up of 34 months [5–15]. This analysis revealed first of all that the majority of the lesions with a mean size of 2.60 cm have a slow growth rate (mean rate 0.28 cm per year) and rarely metastasise and this in only 1% of cases while under active monitoring. Secondly, the initial tumour size did not predict the overall growth rate (*P* = .46). An absolute safe cut-off for surveillance may therefore not exist since the metastatic potential of observed tumours cannot be predicted. Only 46% of the patients had pathological evaluation however and the pathologically analysis was incomplete in many cases.

## 3. NATURE OF SMALL RENAL MASSES IN RELATION TO TUMOUR SIZE

### 3.1. Benign lesions

A significant number of renal masses are actually benign tumours. It remains difficult to differentiate oncocytomas, for instance, from renal cell carcinomas (RCCs) and this even with the most advanced cross-sectional imaging techniques. In a literature review by Chawla et al. [16] including 76 tumours (12% oncocytomas, 88% RCCs), there was no statistical difference in mean initial tumour size (2 versus 2.2 cm) or mean growth rate (0.16 versus 0.35 cm per year) comparing oncocytomas versus RCCs, respectively [16, 17]. Radiographic data alone are yet still unable to predict the exact natural history of small renal masses. A retrospective study by Remzi et al. [19] reported that 81.9% of all small renal masses were RCC and only 17% were correctly defined as benign on preoperative CT.

Although up to 90% of the solid renal masses are RCCs, elderly patients with small renal masses are up to 3.5 times more likely to have benign lesions than RCCs [18]. Recent data suggest that smaller lesions may have an even greater chance of being benign than previously recognised [20–22]. As the tumour size increases, there is a significantly greater probability that the tumour is malignant versus benign, clear cell versus papillary RCC, and high-grade versus low-grade RCC. These results provide a pathological basis for the use of surveillance strategies in the treatment of small renal masses in poor surgical candidates. In the EORTC 30904 study by Van Poppel et al. [21], 11.6% of the 541 surgically removed tumours (≤5 cm) were benign. In another study by Gill et al. [22], 30% of the 100 tumours treated with laparoscopic partial nephrectomy were benign. The tumours had a mean diameter of 2.8 cm.

Schlomer et al. [23] examined the relationship between tumour size and pathological findings in 349 renal masses (Table 1). The percent of malignant tumours increased from 72.1% for those <2 cm in diameter to 93.7% for those >7 cm (odds ratio = 1.39; 95% confidence interval, 1.17–1.65). Lesions ≤4 cm and >7 cm were associated with high-Fuhrman grade (G3/G4) in fewer than 28% and in greater than 63% of the cases, respectively (chi-square test *P* < .001). Small renal tumours are more likely to be benign or to be of lower grade than larger tumours.

It seems to be that small renal masses might be benign and that larger renal masses are RCC. Tumour size alone however does not provide adequate information for deciding on the optimal treatment. Preoperative evaluation should be more refined.

### 3.2. Small renal tumours: how benign are they?

Remzi et al. [24] reviewed data of 287 small renal masses (≤4 cm) detected by CT and treated surgically with pathological analysis. They found no correlation between tumour size and benign histology. In this analysis, tumours were stratified according to preoperative diameter into three or two groups (Table 2). What they found is that Fuhrman grades G3 and G4, higher pathological stage (pT3a or greater), and metastatic disease were seen significantly more frequently in tumours >3 cm in diameter. This difference was however not observed when masses ≤2 cm in diameter were compared with those measuring 2.1–3.0 cm. Taking into account that measuring tumour diameters is difficult and is based on the reliably of sequential imaging, one may speculate that surveillance strategies should be limited to patients with tumours below the diameter of 3 cm.

To analyse which malignant potential small renal masses might have, Gill et al. analyzed their own series. They included tumours with a mean diameter of 2.8 cm. Even though it was assumed that the risk of malignancy was less in smaller tumours, their findings showed that the majority of these tumours were malignant with growth potential [16, 18].

Also, Hsu et al. [25] showed that 38% of the 50 resected RCCs of <3 cm had extracapsular extension (pT3 or pT4) and 28% were Fuhrman grade G3/G4.

Minardi et al. [26] considered 48 patients with pT1a clear cell RCC. Of the patients treated with nephron-sparing surgery, 3.9% died of metastatic renal cancer at a median follow-up of 2 years, with one patient having Fuhrman grade 2 (G2), one having G3 and two having G4 RCCs.

These findings support resection of even small lesions and support that recurrence and death are possible in patients with small renal tumours even with low-grade RCC. Active monitoring in these patients would have been unsafe.

We can conclude that not all small renal tumours are harmless and that even very small lesions may progress to metastatic disease.

## 4. GROWTH RATE

### 4.1. Can the initial tumour size predict its subsequent growth rate?

As stated above, one could not identify a significant correlation between initial tumour size and growth rate in an analysis of 157 tumours from 5 observational series [5, 7–9, 13, 16] (*P* = .46). Therefore, the initial tumour size cannot predict the subsequent growth rate.

### 4.2. Growth or no growth: what can it predict?

When growth of (small) renal masses is apparent, it becomes more likely that these lesions need treatment because malignant behaviour is suspected. A recent meta-analysis could confirm this. The mean growth rate of pathologically confirmed RCC (92%) was significantly greater than for tumours continued under surveillance (0.40 ± 0.36 versus 0.21 ± 0.40 cm per year, *P* = .0001). Also Kouba et al. [1] showed a higher growth rate in patients undergoing eventual intervention than for tumours continued under surveillance (0.90 versus 0.61 cm per year, resp.; *P* = .1486).

It remains however difficult to predict biological behaviour of small renal masses, even if they do not show growth. We cannot conclude that small renal masses that do not show growth during surveillance are less likely to be cancerous.

Kunkle et al. [17] observed 106 renal masses for at least 1 year and compared clinical, radiographic, and pathological characteristics of the lesions with zero or negative radiographic growth (33%) versus those with positive growth (67%) (median, 0.31 cm per year). Rates of malignancy were similar in both groups (83% and 89%, resp.; *P* = .56). The results suggest that a lack of radiographic growth is not associated with malignant potential or pathological findings.

In other observation series, Kouba et al. [1]. observed a significant difference in growth rates between grades 2 and 3 but not between grades 1 and 2 tumours. Growth rate did not correlate with prognosis [16, 17]. In conclusion, the majority of small renal masses grow and the majority are cancer. One cannot safely assume that a lack of growth on serial CT scanning confirms the absence of malignancy. No clinical or radiological predictors of growth rate are yet identified [17].

### 4.3. What about age and growth rate?

A meta-analysis of published observation series [5, 6, 11–13, 16] demonstrated an inverse correlation between increasing age and tumour growth rate. Also in the observation study by Kouba et al. [1], patients ≤60 years (*n* = 15) had more rapid growth rate of renal masses compared with those >60 years (*n* = 31) (0.90 versus 0.60, *P* = .0570). Because younger patients have longer life expectancies and most likely fewer comorbidities, these results provide greater support to propose surgery in young patients with renal masses [1].

## 5. PROGRESSION TO METASTATIC DISEASE

There are no published reports of metastasis occurring in the absence of tumour growth. While all observed lesions do have the potential to metastasise, the risk to do so appears low in the absence of growth. Yet follow-up is however short and we have to take into account the retrospective nature of these studies.

## 6. ROLE OF BIOPSY

The value of tumour biopsies remains controversial. Some histologically proven RCCs may demonstrate nonaggressive behaviour, negative biopsy would not rule out RCC, and a positive biopsy may significantly understage or undergrade the lesion [16, 27, 28]. Preoperative needle biopsies remain inaccurate in 18 to 23% of patients [29, 30]. However, a recent retrospective study of Vasudevan et al. [31] revealed however that a higher than previously anticipated proportion of incidentally detected small renal masses are benign. Given the high sensitivity and specificity of biopsies in their hands, they proposed that there is value in taking a core biopsy of small incidental renal lesions. The investigators concluded that biopsy could avoid unnecessary surgery in one third of the incidental renal masses. When using contemporary biopsy techniques, the risk of tumour seeding or hemorrhage is extremely low [32].

Although the accuracy of fine-needle percutaneous biopsy with CT guidance of small renal masses (<4 cm) evaluated in 88 patients was high for histopathological tumour type (92%), biopsy was less accurate in evaluating Fuhrman grade (70%) [33]. Many benign tumours may be diagnosed with the help of biopsy findings, but more data are still needed to understand the overall accuracy of biopsy for the diagnosis of benign tumours [34].

We currently do not have any reliable molecular marker for separating indolent from aggressive tumours [35]. However a recent pilot study [36] demonstrated that the detection of the MN/CA9 gene can reliably be detected in fine-needle aspiration biopsy and that this gene marker can be helpful in separating malignant from benign renal tumours. It remains to be confirmed that other molecules such as carbonic anhydrase IX and vascular endothelial growth factor could potentially be used in conjunction with usual prognostic parameters for refining prognosis in small renal masses.

## 7. ACTIVE TREATMENT OPTIONS

In the majority of the patients, nephron-sparing surgery remains the gold standard treatment because it is a safe and effective procedure and because even very small lesions may progress to metastatic disease.

For frail patients who are not fit for open or laparoscopic nephron-sparing surgery, treatment option by minimally invasive techniques, such as radiofrequency ablation and cryoablation under ultrasound or CT guidance, might be a middle way between aggressive treatment and expect monitoring. These newer minimally invasive therapies have become available for the treatment of small renal masses. In patients who have medical comorbidities or a limited life expectancy, radio frequency ablation and cryoablation provides reasonable long-term oncological control and it may have a role in the management of small renal masses [37]. Meticulous long-term follow-up is however required in patients receiving radio frequency ablation.

Cryoablation is less well investigated. Schwart et al. [38] published is Urology in 2006 a retrospective analysis of cryoablation of small peripheral renal masses. They concluded that renal cryotherapy is a viable option for nephron-sparing surgery in small, peripheral renal lesions. The procedure has well-tolerated results, may be considered in patients who are not good candidates for open surgical approaches, in minimal morbidity, and has shown encouraging treatment results. Close post treatment surveillance is essential. Meticulous long-term follow-up is however required before these new ablative treatments can be considered a valuable alternative to surgical extirpation.

Furthermore, whether these treatments provide a benefit over surveillance strategies in older, poor surgical candidates with limited life expectancy needs to be addressed in prospective, randomised, clinical trials.

## 8. CONCLUSIONS

Approximately 26–33% of observed small renal masses do not show radiographic growth. Therefore, it has been suggested that a short period of active monitoring may be feasible for a very selected patients group.

It has been proposed to delay treatment in these patients when the tumour does not show growth. However, even though tumour growth might be absent or slow, a proportion of these tumours will express significant malignant behaviour, since the natural history and biological potential of small renal masses can still not be unambiguously predicted at present. We therefore believe that the indications for active surveillance with regular radiographic follow-up are limited to elderly and/or infirm patients with competing health risks, to those with limited life expectancy and to poor surgical candidates.

In all other patients, active surveillance can be considered in the context of a study protocol only. It is noteworthy that, because of the increase in life expectancy even in a 70-year-old otherwise healthy patient, some sort of active treatment of small renal masses should be preferred over surveillance [32]. Nephron-sparing surgery remains the gold standard treatment of (small) renal masses. However, minimally invasive techniques like radiofrequency ablation and cryoablation have emerged. While there is still a need for prospective, randomised, clinical trials with sufficient follow-up, these procedures are considered suitable for patients who have limited life expectancy or are high-risk-surgicalcandidates. These treatment strategies provide reasonable short- and intermediate-term oncological control, however long-term results are unavailable so far.

When facing patients with small bilateral or multiple renal tumours, treatment strategies will not significantly differ compared to patients with small single lesions, since the indications for active surveillance remain limited to elderly and/or infirm patients with competing health risks and those with limited life expectancy.

The value of tumour biopsies remains controversial. Preoperative needle biopsy remains inaccurate in 18 to 23% of patients. Molecular or biochemical markers as well as better imaging techniques are required to select individuals at the highest risk for tumour progression.

To date, there is no a nonstandardised follow-up protocol for surveillance. There is the patients' fear of harbouring a tumour with an uncertain malignant potential. Surveillance requires a high degree of individual compliance by the patient. Even in compliant patients there is a risk that the onset of progression will be missed. When small renal masses have progressed to metastatic disease, there are no effective systemic therapies available. The safety of longer-term surveillance is still questionable. On the basis of current literature, we have no data on the risk and cost of patients on active surveillance.

Finally, the treatment modality of active monitoring should always be combined with close follow-up imaging and should be allowed only when the patient and the urologist accept the calculated risk. Long-term, prospective studies are needed to provide a more accurate assessment of the natural history and metastatic potential of small renal masses in the long term. Until long-term follow-up results including outcome, risk, and cost of patients managed expectantly are available, no definitive guidelines can be established for the surveillance of small renal masses.

## Figures and Tables

**Table 1:** Tumour size versus histology [23].

Tumour size (cm)	No. of benign tumours (%)	No. of RCC (%)
0.0–0.9 (*n* = 7)	1 (14.3)	6 (85.7)
1.0–1.9 (*n* = 54)	16 (29.6)	38 (70.4)
2.0–2.9 (*n* = 83)	19 (22.9)	63 (75.9)
3.0–3.9 (*n* = 63)	11 (17.5)	52 (82.5)
4.0–4.9 (*n* = 32)	3 (9.4)	28 (87.5)
5.0–5.9 (*n* = 29)	2 (6.9)	26 (89.7)
6.0–6.9 (*n* = 18)	0 (0.0)	18 (100.0)
7.0 or greater	4 (6.3)	58 (92.1)
Totals	56 (16.0)	289 (82.8)

**Table 2:** Tumour diameter and aggressiveness [24].

65 tumours: ≤2 cm	103 tumours: 2.1–3.0 cm	119 tumours: 3.1–4.0 cm
73.8% RCC	78.6% RCC	82.4% RCC
24.6% benign	20.4% benign	16.0% benign

168 tumours: ≤3 cm		119 tumours: 3.1–4.0 cm

10.9% pT3a or greater		35.7% pT3a or greater
4.7% G3-G4		25.5% G3-G4
2.4% M+		8.4% M+

## References

[1] Kouba E, Smith A, McRackan D, Wallen EM, Pruthi RS (2007). Watchful waiting for solid renal masses: insight into the natural history and results of delayed intervention. *The Journal of Urology*.

[2] Derweesh IH, Novick AC (2003). Small renal tumors: natural history, observation strategies and emerging modalities of energy based tumor ablation. *The Canadian Journal of Urology*.

[3] Pantuck AJ, Zisman A, Belldegrun AS (2001). The changing natural history of renal cell carcinoma. *The Journal of Urology*.

[4] Lee CT, Katz J, Shi W, Thaler HT, Reuter VE, Russo P (2000). Surgical management of renal tumors 4 cm. Or less in a contemporary cohort. *The Journal of Urology*.

[5] Volpe A, Panzarella T, Rendon RA, Haider MA, Kondylis FI, Jewett MAS (2004). The natural history of incidentally detected small renal masses. *Cancer*.

[6] Rendon RA, Stanietzky N, Panzarella T (2000). The natural history of small renal masses. *The Journal of Urology*.

[7] Fujimoto N, Sugita A, Terasawa Y, Kato M (1995). Observations on the growth rate of renal cell carcinoma. *International Journal of Urology*.

[8] Bosniak MA (1995). Observation of small incidentally detected renal masses. *Seminars in Urologic Oncology*.

[9] Bosniak MA, Birnbaum BA, Krinsky GA, Waisman J (1995). Small renal parenchymal neoplasms: further observations on growth. *Radiology*.

[10] Oda T, Miyao N, Takahashi A (2001). Growth rates of primary and metastatic lesions of renal cell carcinoma. *International Journal of Urology*.

[11] Kassouf W, Aprikian AG, Laplante M, Tanguay S (2004). Natural history of renal masses followed expectantly. *The Journal of Urology*.

[12] Wehle MJ, Thiel DD, Petrou SP, Young PR, Frank I, Karsteadt N (2004). Conservative management of incidental contrast-enhancing renal masses as safe alternative to invasive therapy. *Urology*.

[13] Kato M, Suzuki T, Suzuki Y, Terasawa Y, Sasano H, Arai Y (2004). Natural history of small renal cell carcinoma: evaluation of growth rate, histological grade, cell proliferation and apoptosis. *The Journal of Urology*.

[14] Lamb GWA, Bromwich EJ, Vasey P, Aitchison M (2004). Management of renal masses in patients medically unsuitable for nephrectomy—natural history, complications, and outcome. *Urology*.

[15] Sowery RD, Siemens DR (2004). Growth characteristics of renal cortical tumors in patients managed by watchful waiting. *The Canadian Journal of Urology*.

[16] Chawla SN, Crispen PL, Hanlon AL, Greenberg RE, Chen DYT, Uzzo RG (2006). The natural history of observed enhancing renal masses: meta-analysis and review of the world literature. *The Journal of Urology*.

[17] Kunkle DA, Crispen PL, Chen DYT, Greenberg RE, Uzzo RG (2007). Enhancing renal masses with zero net growth during active surveillance. *The Journal of Urology*.

[18] Rendon RA, Jewett MAS (2006). Expectant management for the treatment of small renal masses. *Urologic Oncology*.

[19] Remzi M, Katzenbeisser D, Waldert M (2007). Renal tumour size measured radiologically before surgery is an unreliable variable for predicting histopathological features: benign tumours are not necessarily small. *BJU International*.

[20] Frank I, Blute ML, Cheville JC, Lohse CM, Weaver AL, Zincke H (2003). Solid renal tumors: an analysis of pathological features related to tumor size. *The Journal of Urology*.

[21] Van Poppel H, Da Pozzo L, Albrecht W (2007). A prospective randomized EORTC intergroup phase 3 study comparing the complications of elective nephron-sparing surgery and radical nephrectomy for low-stage renal cell carcinoma. *European Urology*.

[22] Gill IS, Matin SF, Desai MM (2003). Comparative analysis of laparoscopic versus open partial nephrectomy for renal tumors in 200 patients. *The Journal of Urology*.

[23] Schlomer B, Figenshau RS, Yan Y, Venkatesh R, Bhayani SB (2006). Pathological features of renal neoplasms classified by size and symptomatology. *The Journal of Urology*.

[24] Remzi M, Özsoy M, Klingler H-C (2006). Are small renal tumors harmless? Analysis of histopathological features according to tumors 4 cm or less in diameter. *The Journal of Urology*.

[25] Hsu RM, Chan DY, Siegelman SS (2004). Small renal cell carcinomas: correlation of size with tumor stage, nuclear grade, and histologic subtype. *American Journal of Roentgenology*.

[26] Minardi D, Lucarini G, Mazzucchelli R (2005). Prognostic role of Fuhrman grade and vascular endothelial growth factor in pT1a clear cell carcinoma in partial nephrectomy specimens. *The Journal of Urology*.

[27] Campbell SC, Novick AC, Herts B (1997). Prospective evaluation of fine needle aspiration of small, solid renal masses: accuracy and morbidity. *Urology*.

[28] Dechet CB, Zincke H, Sebo TJ (2003). Prospective analysis of computerized tomography and needle biopsy with permanent sectioning to determine the nature of solid renal masses in adults. *The Journal of Urology*.

[29] Johnson PT, Nazarian LN, Feld RI (2001). Sonographically guided renal mass biopsy: indications and efficacy. *Journal of Ultrasound in Medicine*.

[30] Permpongkosol S, Link RE, Solomon SB, Kavoussi LR (2006). Results of computerized tomography guided percutaneous ablation of renal masses with nondiagnostic pre-ablation pathological findings. *The Journal of Urology*.

[31] Vasudevan A, Davies RJ, Shannon BA, Cohen RJ (2006). Incidental renal tumours: the frequency of benign lesions and the role of preoperative core biopsy. *British Journal of Urology*.

[32] Jewett MAS, Stöckle M (2006). The motion: surveillance is an option for renal cancer. *European Urology*.

[33] Neuzillet Y, Lechevallier E, Andre M, Daniel L, Coulange C (2004). Accuracy and clinical role of fine needle percutaneous biopsy with computerized tomography guidance of small (less than 4.0 cm) renal masses. *The Journal of Urology*.

[34] Silverman SG, Gan YU, Mortele KJ, Tuncali K, Cibas ES (2006). Renal masses in the adult patient: the role of percutaneous biopsy. *Radiology*.

[35] Patard J-J (2007). With increasing minimally invasive options for small renal tumours, it is time to develop patient-specific treatment strategies. *European Urology*.

[36] Li G, Cuilleron M, Cottier M (2006). The use of MN/CA9 gene expression in identifying malignant solid renal tumors. *European Urology*.

[37] Levinson AW, Su L-M, Agarwal D (2008). Long-term oncological and overall outcomes of percutaneous radio frequency ablation in high risk surgical patients with a solitary small renal mass. *The Journal of Urology*.

[38] Schwartz BF, Rewcastle JC, Powell T, Whelan C, Manny T, Vestal JC (2006). Cryoablation of small peripheral renal masses: a retrospective analysis. *Urology*.

[39] Finley DS, Beck S, Box G (2008). Percutaneous and laparoscopic cryoablation of small renal masses. *The Journal of Urology*.

